# Single-Cell NGS-Based Analysis of Copy Number Alterations Reveals New Insights in Circulating Tumor Cells Persistence in Early-Stage Breast Cancer

**DOI:** 10.3390/cancers12092490

**Published:** 2020-09-02

**Authors:** Tania Rossi, Giulia Gallerani, Davide Angeli, Claudia Cocchi, Erika Bandini, Pietro Fici, Michele Gaudio, Giovanni Martinelli, Andrea Rocca, Roberta Maltoni, Francesco Fabbri

**Affiliations:** 1 Biosciences Laboratory, Istituto Scientifico Romagnolo per lo Studio e la Cura dei Tumori (IRST) IRCCS, 47014 Meldola, Italy; giulia.gallerani@irst.emr.it (G.G.); claudia.cocchi@irst.emr.it (C.C.); erika.bandini@irst.emr.it (E.B.); pietrofici@gmail.com (P.F.); francesco.fabbri@irst.emr.it (F.F.); 2 Unit of Biostatistics and Clinical Trials, Istituto Scientifico Romagnolo per lo Studio e la Cura dei Tumori (IRST) IRCCS, 47014 Meldola, Italy; davide.angeli@irst.emr.it; 3 Pathology Unit, AUSL Romagna, Morgagni-Pierantoni Hospital, 47121 Forlì, Italy; michele.gaudio@auslromagna.it; 4 Scientific Directorate, Istituto Scientifico Romagnolo per lo Studio e la Cura dei Tumori (IRST) IRCCS, 47014 Meldola, Italy; giovanni.martinelli@irst.emr.it; 5 Department of Medical Oncology, Istituto Scientifico Romagnolo per lo Studio e la Cura dei Tumori (IRST) IRCCS, 47014 Meldola, Italy; andrea.rocca@irst.emr.it (A.R.); roberta.maltoni@irst.emr.it (R.M.)

**Keywords:** CTCs, breast cancer, single-cell analysis, CNA, NGS, liquid biopsy

## Abstract

**Simple Summary:**

Circulating tumor cells (CTCs) are crucial for the identification of patients with a higher risk of relapse, including those diagnosed with breast cancer (BC). The aim of this study was to explore their molecular aspects in 11 early-stage BC patients during patient management, focusing on copy number alterations (CNAs) and exploiting a single-CTC next-generation sequencing approach. CTCs showed different degrees of aberration based on access time. Moreover, CTCs, in particular those persisting even months after tumor resection, shared CNAs with matched tumor tissue. Enrichment analyses of CNAs on CTCs highlighted peculiar aberrations, especially associated with interferon (IFN)-associated terms. The study of CTCs CNAs can provide information about the molecular mechanisms involving CTC-related processes and their survival ability in occult niches, supporting the goal of exploiting their application in patients’ surveillance and follow-up.

**Abstract:**

Circulating tumor cells (CTCs) are a rare population of cells representing a key player in the metastatic cascade. They are recognized as a validated tool for the identification of patients with a higher risk of relapse, including those diagnosed with breast cancer (BC). However, CTCs are characterized by high levels of heterogeneity that also involve copy number alterations (CNAs), structural variations associated with gene dosage changes. In this study, single CTCs were isolated from the peripheral blood of 11 early-stage BC patients at different time points. A label-free enrichment of CTCs was performed using OncoQuick, and single CTCs were isolated using DEPArray. Libraries were prepared from single CTCs and DNA extracted from matched tumor tissues for a whole-genome low-coverage next-generation sequencing (NGS) analysis using the Ion Torrent S5 System. The analysis of the CNA burden highlighted that CTCs had different degrees of aberration based on the time point and subtype. CTCs were found even six months after surgery and shared CNAs with matched tumor tissue. Tumor-associated CNAs that were recurrent in CTCs were patient-specific, and some alterations involved regions associated with BC and survival (i.e., gains at 1q21-23 and 5p15.33). The enrichment analysis emphasized the involvement of aberrations of terms, associated in particular with interferon (IFN) signaling. Collectively, our findings reveal that these aberrations may contribute to understanding the molecular mechanisms involving CTC-related processes and their survival ability in occult niches, supporting the goal of exploiting their application in patients’ surveillance and follow-up.

## 1. Introduction

Circulating tumor cells (CTCs) constitute the leukemic phase of a solid tumor. They have the potential to give rise to detectable metastasis [1] and to date the major cause of death in cancer patients [2]. Their identification and subsequent molecular analysis, in particular at a single-cell level, provide information about the prognosis and could be useful for a selective choice for targeted therapies and therapy monitoring [3,4,5,6], enforcing the rationale of using CTCs in the clinical application of liquid biopsy approaches.

The only Food and Drug Administration (FDA)-approved CTC assay applied in a clinical setting with a prognostic purpose for several advanced tumors in BC consists in the detection and enumeration of CTCs using the CellSearch system [7]. To date, BC is estimated to be, among the various types of cancer, the most susceptible one to develop recurrences at both the loco-regional and systemic levels, even after decades. Besides mammography and other instrumental tests based on symptom occurrence, there are no recommended tests in the follow-up of operated asymptomatic patients [8,9,10,11]. CTC detection in both early-stage and metastatic BC patients was proven to discriminate cases with a high risk of relapse [6,12,13,14], representing an innovative non-invasive tool to improve BC disease progression monitoring. However, the blunt CTC enumeration does not provide any information about their molecular features. 

High levels of intra- and inter-patient heterogeneity at the genetic and transcriptional levels were described in CTCs isolated from both metastatic and early-stage BC patients [15,16]. In addition to the great genomic heterogeneity derived from somatic variants in cancer-associated genes, a high variability was found in relation to the spectrum of CTCs’ somatic copy number aberrations (CNAs), i.e., structural variations associated with changes in gene expression [16,17]. Few studies have explored the role of CNAs in the CTCs of non-metastatic and metastatic BC patients [18,19,20], but their evolution steps during patients’ clinical management are still not fully elucidated.

In this pilot study, by exploiting a whole-genome low-coverage next-generation sequencing (NGS) approach, we evaluated the CNA profiles of single CTCs isolated from early-stage BC patients at different time points. Analyses of the CNA burden highlighted that CTCs showed different degrees of aberration based on time access and subtype. Moreover, our results revealed that CTCs, in particular those persisting even months after tumor resection, shared CNAs with matched tumor tissue. Enrichment analyses of CNAs on CTCs highlighted peculiar aberrations, especially associated with interferon (IFN)-associated terms. Our work paves the way for new directions with more in-depth studies of the mechanisms related to the field of CTCs and their biology. Our results can lay the groundwork for reinforcing the hypothesis of a pivotal prognostic role of CTCs, useful for applications in long-term surveillance and patient follow-up, bringing out CTCs as an innovative alternative to more conventional and studied approaches.

## 2. Results

### 2.1. Isolation of CTCs

Blood sampling was performed at three time points: one day before surgery (A), one month after surgery (B) and after adjuvant therapy/six months after surgery (C). After enrichment, all the identified CTCs were sorted by the DEPArray platform. We performed downstream analyses on all of the 49 identified single CTCs, as described in Table 1.

We observed that all the patients but one (90.9%) had CTCs at diagnosis, whereas 8 (72.7%) and 7 out of 11 (63.3%) had CTCs at time points B and C, respectively. Interestingly, we observed a 45.8% decrease in the CTC number between time points A (*n* = 24) and B (*n* = 13). We did not notice a significant decrease in the CTC number between time points B (*n* = 13) and C (*n* = 12). In particular, CTCs were persistent six months after surgery (time point C), and we decided to consider them as long-persisting cells without a short-term metastatic potential, since none of the seven patients displaying CTCs at this time point gave any evidence of a disease to date, more than five years after surgery. 

### 2.2. Single-Cell CNA Analysis of CTCs with Whole-Genome NGS 

We performed single-cell molecular analyses to characterize the CNA profile of each single cell. Furthermore, to obtain information regarding the CNA burden, intended as a measure of the degree of genomic alteration at the whole genome level [20], we calculated the Jaccard Index (JI). JI is a measure of similarity, which we computed as the ratio of shared-to-all aberrations between single CTCs and lymphocytes, which we considered as a reference normal chromosomal set.

**Figure 1:**
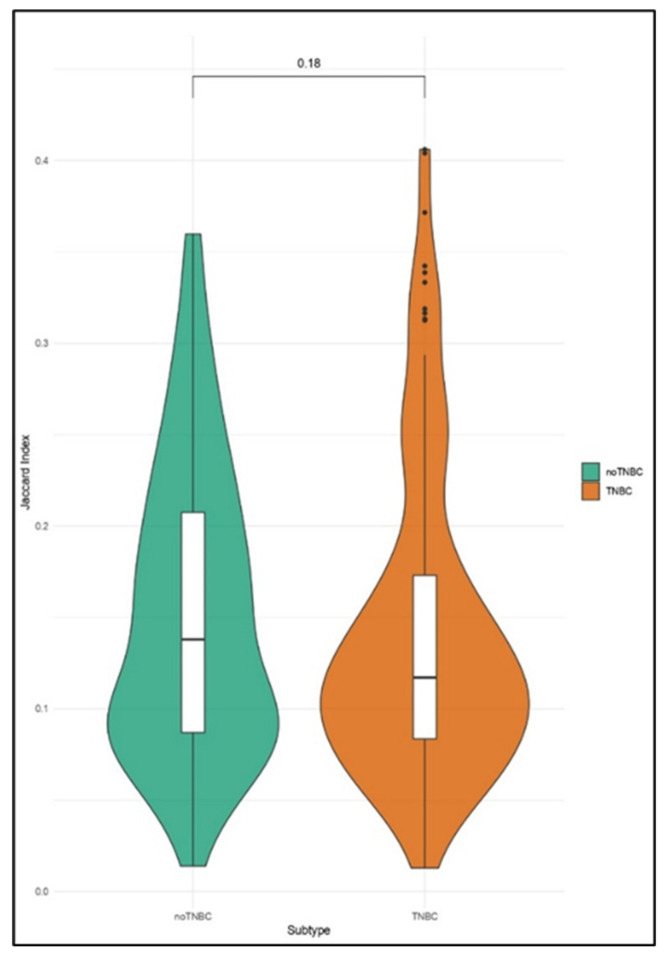
Violin plots showing the distribution of the Jaccard Indexes (JIs) computed by comparing the copy number alteration (CNA) profile of single circulating tumor cells (CTCs) and controls in non-triple negative breast cancer (TNBC) against TNBC.

We observed that almost all CTCs had a JI below 0.4, meaning that the analyzed cells had less than 40% genomic similarity when compared to the reference. The CNA burden of single CTCs of TNBC (*n* = 26) and non-TNBC (*n* = 23) patients did not differ significantly (*p* = 0.18), although the violin plots showed a trend of heterogeneity in terms of the CNA burden (Figure 1). 

The non-TNBC cohort displayed a higher dispersion of JI values of CTCs, suggesting an almost bimodal trend of the violin plot. Conversely, the violin plot of the TNBC group showed that the JIs of CTCs were concentrated at a lower JI median value, implying the presence of CTCs with a less heterogeneous, but greater, CNA burden. 

**Figure 2:**
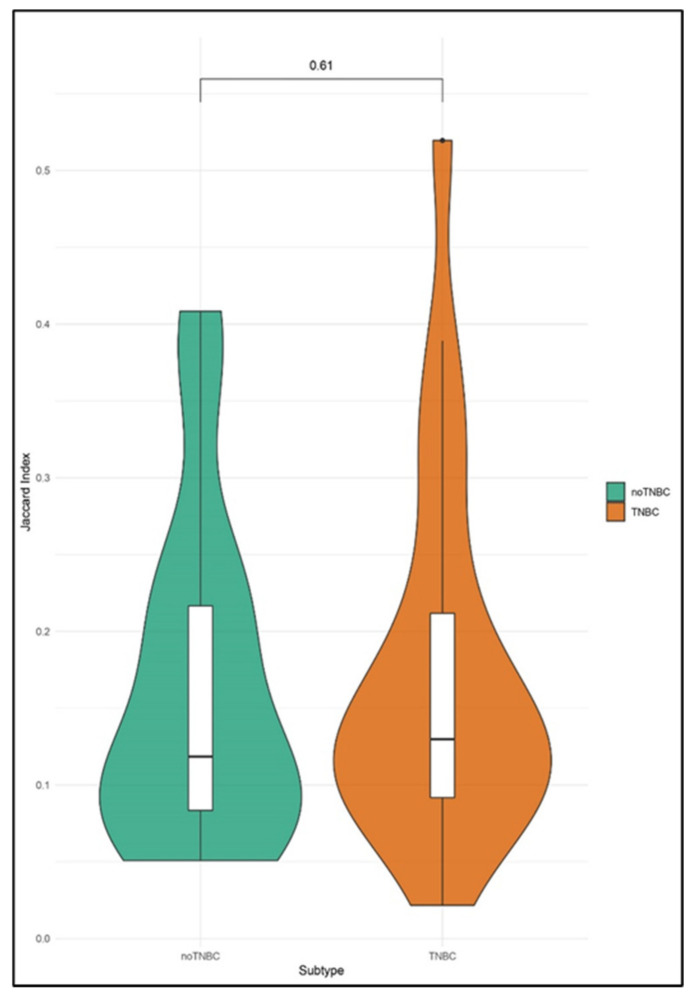
Violin plots showing the distribution of the Jaccard Indexes (JIs) computed by comparing the copy number alteration (can) profile of single circulating tumor cells (CTCs) and matched tumor tissue in non-triple negative breast cancer (TNBC) against TNBC.

By comparing the CNA profiles of CTCs and the correspondent tumor tissues (Figure 2), JIs ranged between 0.02 and 0.52, implying a similarity between CTCs and matched tumor tissues in terms of CNAs between 2% and 52%.

The violin plot of the non-TNBC cohort maintained a bimodal trend, with the CTCs’ CNA profile being heterogeneously different from the primary tumor. Conversely, all of the CTCs of the TNBC cohort differed from the tumor mass in the same manner, since the JIs were located in a concentrated peak. We did not observe any statistical significance between the two cohorts.

**Figure 3:**
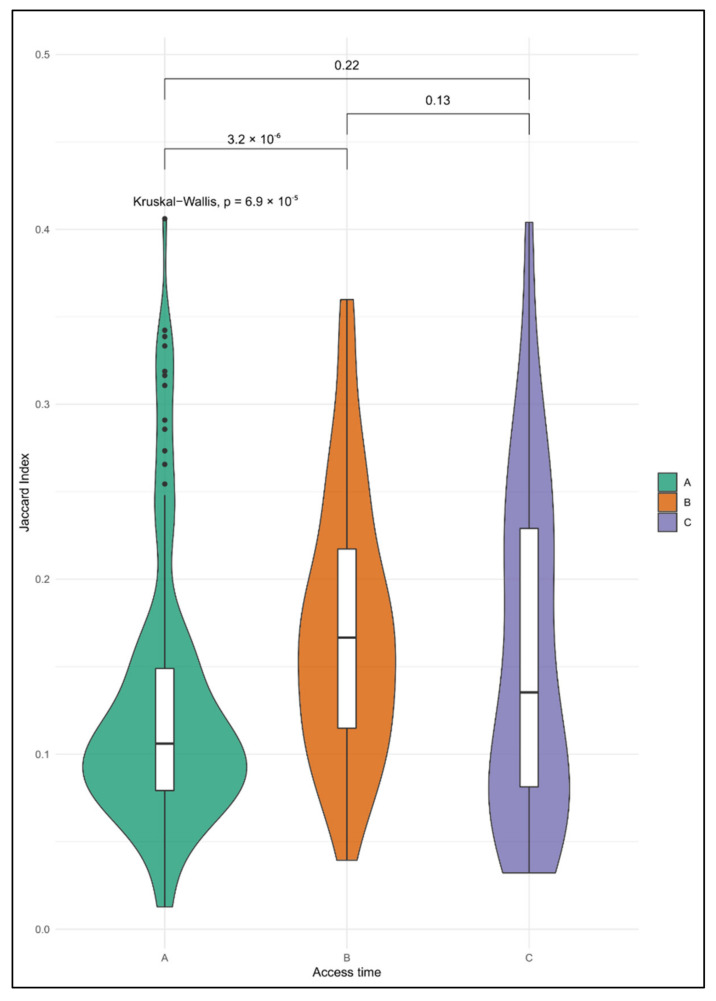
Violin plots showing the distribution of the Jaccard Indexes (JIs) computed by comparing the copy number alteration (can) profile of single circulating tumor cells (CTCs) and control at the access times (**A**) (*n* = 24), (**B**) (*n* = 13) and (**C**) (*n* = 12).

Concerning the CNA burden of single CTCs isolated at each different time point, Figure 3A (*n* = 24), Figure 3B (*n* = 13), and Figure 3C (*n* = 12), we found that the JIs of cells from times A and B differed significantly (*p* = 3.2 × 10^−6^ with Mann–Whitney *U* test). Cells from time B showed a median JI that was slightly higher than that observed at time A, suggesting that surgery (primary tumor excision) and time may select CTCs with a higher and more heterogeneous CNA burden. 

**Figure 4:**
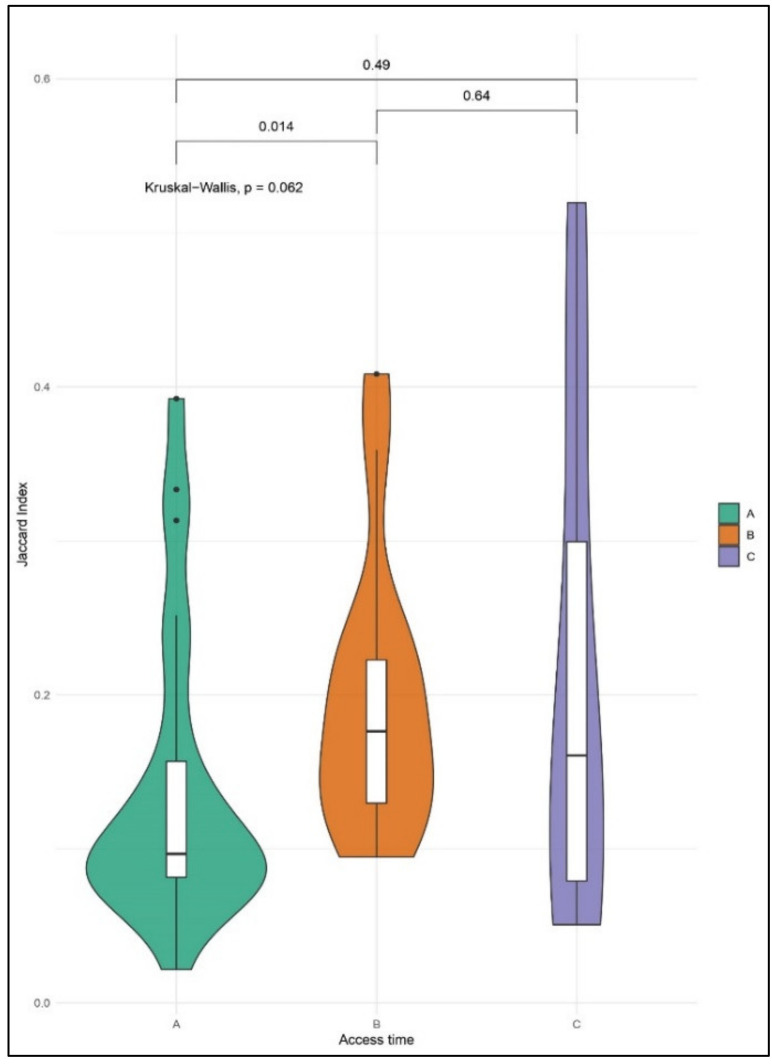
Violin plots showing the distribution of the Jaccard Indexes (JIs) computed by comparing the copy number alteration (CNA) profile of single circulating tumor cells (CTCs) and matched tumor tissue at the access times (**A**) (*n* = 24), (**B**) (*n* = 13) and (**C**) (*n* = 12).

Interestingly, the difference between the median JIs pre- and post-surgery was also shown to be significant by comparing the CNA profile of CTCs and matched tumor tissues (Figure 4). These data suggest that, besides the selection of CTCs with a higher CNA burden, at time point B we observed CTCs that were more similar to the matched primary tumor.

Moreover, although the JIs of CTCs from time point C did not significantly differ when compared to other groups, in both Figure 3 and Figure 4 one can see that the presence of CTCs with more dispersed CNA burdens appeared after adjuvant therapy. In particular, Figure 4 shows that, within the cohort of CTCs of time C, there were cells with a CNA profile similar to the original tumor, which was resected six months before. 

Generally, the reported violin plots illustrate that the CNA burden of CTCs became more dispersed during the disease.

### 2.3. Identification of Aberrations Shared Between CTCs and Matched Tumor Tissue

We wanted to verify the presence of CNAs shared between CTCs and matched tumor tissue, and to understand if shared regions were recurrent along the course of the patients’ disease, in particular in CTCs from time C. 

To accomplish this task, besides single CTC sequencing, we improved the protocol of the Ampli1 LowPass for Ion Torrent (Menarini Silicon Biosystems) to obtain CNA profiles of the tissue starting from FFPE specimens, and we searched for altered common regions at the intra-patient level for 10 patients. 

For five out of six patients who had detectable CTCs at time C, CNA profiling of the FFPE specimen was possible. Interestingly, despite the high CNA burden of CTCs, we detected CNA regions that were shared among tumor tissue and CTCs from times A, B and C. In Table 2, we report the tumor-associated CNA found in at least two CTCs of the same patient and recurrent in cells from time C. Each CTC was codified as follows: PXX (patient code) A/B/C (timepoint)_Cell ID.

All the reported CNAs were chromosomal gains. Six regions were shown to be altered at all time points, eight between the CTCs of time points B and C, while thirteen were shared between those of A and C. Only four regions were altered in the tissue and at the access time C only. Interestingly, TNBC Patient 04 had the highest number of regions in common between matched tissue and CTCs, whereas the CTCs of TNBC Patient 02 only displayed two regions in common with the tissue. Patients 02 and 04 were diagnosed with grade 3 and Ki-67 85% and 80% BC, respectively, but only patient 04 showed vascular invasion. Concerning non-TNBC patients, we observed only one region in the CTCs that was in common with the tissue for each patient. 

Generally, we observed that, beyond persisting after surgery and adjuvant therapy, CTCs from timepoint C had certain aberrations similar to the tumor tissue. All the aberrations were patient-specific. Importantly, among all the alterations, we found some chromosomal regions that have been described in the literature as being involved with BC, as reported in Table 2.

In Table S1, we reported the CNAs shared between CTCs and matched tumor tissue in the whole case series, including for patients who did not display CTCs at time C.

### 2.4. Enrichment Analyses of CNA in Single CTCs

To gain further insights into the function of identified CNAs in single CTCs, we performed enrichment analyses. The results of the enrichment analyses and CTCs are reported in Table S2. Table 3 reports the list of the significantly enriched terms (adjusted *p* < 0.05) of the GO-Molecular Function database.

The most enriched term, found in 10 single CTCs out of 49 (20.4%), was associated with the activation of natural killer (NK) cells in the immune response (GO:0002323). However, the major part of the enriched terms was linked to the activation and proliferation of immune cells (B-cells, NK, lymphocyte), as well as to the response to double-strand RNA (dsRNA) and the phosphorylation of the Signal Transducer and Activator of Transcription (STAT) protein. Interestingly, genes associated with IFN were reported in nearly all of the enriched terms, suggesting a possible involvement in CTC biology.

Collectively, these data indicate that the observed chromosomal aberrations in CTCs seem to occur in regions where genes codify for proteins associated with an immune response. 

## 3. Discussion

In this pilot study, we explored the molecular aspects of CTCs in early-stage BC patients during the disease and after treatments, focusing on the CNAs.

The analysis of the CNA burden showed that CTCs had different degrees of aberrations based on the time point. In particular, we observed that surgery and time may play a role in a sort of selection for almost half of CTCs, targeting those with a higher CNA burden. Indeed, at time B, cells showed a lower CNA burden when compared to time-A CTCs. At the same time, the CNA degree of time C-CTCs, recovered six months after surgery, gets even more heterogeneous, with cells showing a lower median CNA burden than for other time points. Our data might suggest that CTCs with higher levels of aberrations can be more susceptible to adjuvant therapy. The chromosomal status in BC has already been reported in the literature as potentially predicting the response to distinct therapeutic agents, such as anthracyclines [26]. Furthermore, similarity analyses revealed that, among time C-CTCs, some cells resembled the matched tumor more than those from other timepoints, suggesting the presence of a reservoir pouring cells into the blood stream. Although it was not possible to discern where these CTCs came from, it would be interesting to explore if these cells persist in a dormant status within a niche, e.g., the bone marrow [1,27]. Accordingly, CTCs were shown to be present in the peripheral blood of patients with non-Tetastatic BC even after two to five years after surgery, as reported by Trapp et al. and Sparano et al. [28,29]. Moreover, the meta-analysis published by the Early Breast Cancer Trialists’ Collaborative Group (EBCTCG) highlighted that relapse may occur at a constant rate from five to 20 years, also in patients with small tumors (T1) and with negative axillary lymph nodes (N0) [30,31]. These recent data, in addition to our results, may suggest that late recurrence is due to micrometastases that spread CTCs genomically similar to the primary tumor. Hence, the detection and, in particular, molecular characterization of CTCs in early-stage BC may contribute to the decision-making of clinicians in the selection of patients to strictly follow up and to evaluate for secondary adjuvant treatments [32].

Consistent with this, we found out that several aberrations were commonly present in primary tumor tissue and CTCs from time C, suggesting the presence of regions potentially associated with their persistence. It would be interesting to understand if these CNAs could act as a marker or cause of tumor cell persistence. Remarkably, some regions have been reported to be typically present in BC. For instance, gains in 1q have been characterized in BC as being complex and discontinuous alterations, with the major part of the amplifications involving the regions 1q21–q22, 1q23–q24, 1q32 and 1q42–q44 [23,24]. Furthermore, among the recurrent gains in CTCs of BC patients, Kanwar et al. reported the amplification of two regions in 1q21-23 (154′963’903-155’224’815; 155’247’948-156’217’829) with tumorigenic functions associated with various mechanisms, including invasion and metastasis [27]. Importantly, the 1q21-23 region is nearly comparable to the gain we observed in the cancer tissue and CTCs of Patient 4 involving regions 1q23.1, 1q23.2 and 1q23.3. At the same time, another aberration, observed to be recurrent in the CTCs and matched tumor tissue of Patient P04, overlaps the region 5p15.33. This region, which harbors the telomerase reverse transcriptase (TERT) gene, was described as a chromosomal gain and found to be associated with a negative outcome in BC [21]. TERT is involved in avoiding telomere loss, hence preventing apoptosis and senescence of malignant cells, and it has been associated with a higher risk of disease recurrence or death in BC patients [33]. Thus, alterations in these regions may be involved in and may contribute to the persistence of CTCs, maintaining a tumor-associated CNA. Notably, these aberrations are patient-specific, as we did not observe any recurrent tumor-associated-CNA at the interpatient level. These data suggest the presence of a multitude of pathways behind molecular mechanisms associated with CTC persistence, confirming the heterogeneity of this cell population. 

Despite this study being conducted on a limited case series, our findings highlight the contribution of CTCs’ CNAs in the elucidation of molecular mechanisms of CTC biology, focusing on their ability to survive in an occult reservoir. 

Although we observed a great CNA heterogeneity, enrichment analyses revealed that type I interferon (IFN)-associated genes were thoroughly altered in CTCs, suggesting an involvement of their pathway in tumor spreading and metastatic cascade. IFN has been shown to have an elusive role. Indeed, although a major activation of the IFN pathway was reported to have an apoptosis- and senescence-promoting capability, this actor was shown to also be implied in migratory and therapy-resistance abilities in primary inflammatory BC [34,35,36]. Hence, it might be of interest to deepen whether IFN-associated genes are involved in mechanisms of CTC survival.

## 4. Materials and Methods 

### 4.1. Patients

Eleven patients diagnosed with early-stage BC were enrolled between 2013 and 2014. The clinical-pathological characteristics are listed in Table S3. Peripheral whole blood was collected at three time points: one day before surgery (A), one month after surgery (B) and after adjuvant therapy/six months after surgery (C). None of the patients underwent neoadjuvant therapy or had detectable metastasis at diagnosis. Patients’ follow-up was conducted following the international guidelines, which recommend mammography and instrumental tests based on symptoms’ occurrence. Tumor tissues were formalin-fixed paraffin-embedded (FFPE), obtained from the U.O. Anatomia Patologica, G.B. Morgagni-L. Pierantoni hospital in Forlì. The FFPE specimens were available for 10 patients. All subjects gave written informed consent to the conservation and use of the samples for research purposes. The study was conducted in accordance with the Declaration of Helsinki, and the protocol was approved on 24 October 2012 by the Romagna Ethics Committee (CEROM) of Meldola (IRSTB008).

### 4.2. CTC Enrichment and Isolation at The Single-Cell Level From Peripheral Whole Blood 

CTCs were enriched from approximately 20 mL of whole blood by using OncoQuick (Greiner Bio-One GmbH, Frickenhausen, Germany), following the manufacturer’s instructions. Enriched CTCs were then fixed with 4% paraformaldehyde and incubated with anti-EpCAM and anti-pan-cytokeratin (CK) antibodies (CTC markers), anti-CD45 (leukocyte marker) and 4′,6-diamidino-2-phenylindole (DAPI) for nucleus staining. Samples were resuspended and loaded with SB115 manipulation buffer (Menarini Silicon Biosystems, Castel Maggiore, Italy) into A300K cartridges, and the analyses and isolation were carried out with DEPArray v1 (Menarini Silicon Biosystems). CTCs (DAPI^+^/EpCAM^+^/CK^+^/CD45^−^) and control lymphocytes (DAPI^+^/CD45^+^) were collected as pure single cells in 0.2 µL tubes. Following a phosphate-buffered saline (PBS) wash and volume reduction, single cells were subjected to whole genome amplification (WGA) by using the Ampli1™ WGA Kit (Menarini Silicon Biosystems), with a lysis step performed overnight in the thermal cycler and the others steps following the protocol provided by the manufacturer. The presence of amplified DNA was assessed by employing the Ampli1™ QC Kit (Menarini Silicon Biosystems). The products of PCR were then run on a 2% agarose gel and visualized using Chemidoc (BioRad, Hercules, CA, USA). 

### 4.3. DNA Extraction From FFPE Specimens

Tumoral areas were scraped from 10 µm slides, and the AllPrep FFPE DNA/RNA kit (Qiagen, Hilden, Germany) was used, following the protocol provided by the manufacturer. For the CNA analyses, extracted DNA was subjected to WGA with the Ampli1™ WGA Kit (Menarini Silicon Biosystems) like for the single cells in order to align the downstream analyses, but avoiding the cell lysis step. 

### 4.4. Library Preparation and Whole-Genome Low-Coverage Sequencing

The presence of CNA was investigated with a whole-genome low-coverage NGS approach by using the Ampli1™ LowPass Kit for the Ion Torrent (Menarini Silicon Biosystems) workflow described by Ferrarini et al. [37]. The size selection of the pooled libraries was performed using E-Gel™ SizeSelect™ II Agarose Gels, 2% (Invitrogen, Carlsbad, CA, USA) on E-Gel™ iBase™ and E-Gel™ Safe Imager™ (Invitrogen). The concentration and length of the pool were assessed using the Qubit™ 3.0 Fluorometer (Invitrogen) and Bioanalyzer High Sensitivity DNA Kit (Agilent Technologies, Waldbronn, Germany), respectively. The pooled libraries were loaded on an Ion 520™ Chip (Thermo Fisher Scientific, Waltham, MA, USA). Template preparation on Ion Chef and sequencing on the GeneStudio™ S5 System (Thermo Fisher Scientific) were performed, setting the run as described in the Ampli1 LowPass protocol.

### 4.5. Bioinformatic Analyses and Statistical Analyses

Starting from the raw data (BAM files) of the samples derived by Ion GeneStudio™ S5 runs, we developed a customized pipeline that included different steps. At first, we called CNAs using Control-FREEC [38]. Two different non-parametric tests (Mann–Whitney and Kolmogorov–Smirnov) were used on the Control-FREEC results to assess the statistical significance of each CNA call. CNAs where at least one of the two *p*-values was greater than 0.05 were filtered out.

We computed the Jaccard index (JI), a measure of similarity in terms of CNAs, comparing each CTC against two reference sets: a set of CNAs derived by different lymphocytes and a set of CNAs derived by 10 matched tissue samples. JI is computed as the size of the intersection of CNAs among the compared samples divided by their union:

$$J\left(A,B\right)=\frac{A\cap B}{A\cup B}$$

where *A* is the set of CNAs of the CTC sample, and *B* is the set of CNAs of a reference sample (a lymphocyte or the matched tumor tissue). Then, we built violin plots, grouping CTCs based on two different features: timepoint and BC subtype (TNBC or non-TNBC).

For each group, we evaluated the statistical differences against the other groups using two non-parametric tests. For access time separations in which more than two CTC groups were present, we applied the Kruskal–Wallis test in order to assess the equableness of the group medians. Finally, for each group pair in each condition, we applied the Mann–Whitney *U* test. 

We performed enrichment analyses of the CNAs observed in single CTCs to discover significantly enriched terms based on the alterations found at the level of genes included in terms listed in different databases. For each significant CNA, we extracted at least 50% of the length of all the genes located inside it. On the basis of this gene list, for each sample, we performed an enrichment analysis on Gene Ontology (GO) datasets (Biological Process, Cellular Component and Molecular Function) [39], Kyoto Encyclopedia of Genes and Genomes (KEGG) [40] and Reactome [41] using enrichR [42], and we filtered out all the terms with an adjusted *p*-value lower than 0.05.

## 5. Conclusions

Taken together, our data suggest that the presence of tumor-associated CNAs in CTCs isolated in patients six months after surgery may provide new insights into understanding the molecular mechanisms associated with persistence and disease relapse. The study of the pathways associated with IFN may unravel the processes of migration and immune-escape of CTCs. Finally, these results may pave the way for innovative patient surveillance approaches, aiming at individuating those at risk of earlier metastasis.

## Figures and Tables

**Table 1:** Number of CTCs isolated for each patient at each access time A, B and C.

Patient Code	BC Subtype	Access Time			Total
		A	B	C	
1	TNBC	6	0	0	6
2	TNBC	1	2	1	4
3	TNBC	0	1	3	4
4	TNBC	6	3	3	12
TNBC total		13	6	7	26
6	HER2+	1	1	1	3
7	Luminal A	1	1	1	3
8	Luminal A	2	3	0	5
9	Luminal A	1	1	1	3
10	Luminal A	1	0	2	3
11	Luminal A	3	0	0	3
12	Luminal A	2	1	0	3
Non-TNBC total		11	7	5	23
Total		24	13	12	49

BC: breast cancer; TNBC: triple-negative BC; HER2: H uman Epidermal Growth Factor Receptor 2.

**Table 2:** List of the altered regions in tumor tissue and in at least two CTCs of the same patient isolated at all access times.

Chromosome:Start–End (bp)	Region/s	CTC ID Timepoint A	CTC ID Timepoint B	CTC ID Timepoint C	Reference
Patient P02					
16:81368788–82403137	16q23.3	P02A__2	P02B_1	P02C_1, P02C_2	
21:40355250–43442658	21q22.2, 21q22.3	-	P02B_1	P02C_1	[21]
Patient 04					
1:33248192–58929280	1p35.1, 1p34.3, 1p34.2, 1p34.1, 1p33, 1p32.3, 1p32.2	P04A_5	-	P04C_3	
1:156891657–162239674	1q23.1, 1q23.2, 1q23.3	P04A_2, P04A_5	P04B_2	P04C_1, P04C_3	[19,22,23,24]
1:162239674–167163992	1q23.3, 1q24.1	-	P04B_1	P04C_1	[19,22,23,24]
1:231729524–234612196	1q42.2	-	-	P04C_1, P04C_3	[22,23,24]
2:36278338–54693738	2p22.2, 2p22.1, 2p21, 2p16.3, 2p16.2	P04A_6	-	P04C_1, P04C_3	
3:72930104–74214062	3p13	-	-	P04C_1, P04C_3	
5:0–2025694	5p15.33	P04A_7, P04A_9	-	P04C_3	[21,25]
6:19336170–21177710	6p22.3	-	-	P04C_1, P04C_3	
7:36023910–36646646	7p14.2	04C458_7	-	P04C_3	
7:151606990–156715054	7q36.1, 7q36.2, 7q36.3	-	P04B_2	P04C_1	
8:92927068–94286848	8q21.2	P04A_8	-	P04C_1, P04C_3	
8:121094607–123567334	8q24.12, 8q24.13	P04A_9	-	P04C_3	
9:109019168–109755681	9q31.2	04A459_9	-	P04C_1	
10:124680720–129276108	10q26.13	P04A_6	-	P04C_3	
11:17862938–20072786	11p15.1	P04A_2	-	P04C_1	
11:112838832–119700100	11q23.2, 11q23.3	P04A_2, P04A_5		P04C_1	
13:24308328–24860790	13q12.12	-	-	P04C_1, P04C_3	
14:75318986–75871448	14q24.3	P04A_9	-	P04C_1	
14:94448360–96128388	14q32.13	-	P04B_2	P04C_1, P04C_3	
15:80279738–85999918	15q25.1, 15q25.2, 15q25.3	P04A_2, P04A_5	-	P04C_3	
16:84526686–87288996	16q24.1	P04A_5	P04B_1, P04B_2, P04B_3	P04C_1	
17:17494630–19520324	17p11.2	-	P04B_3	P04C_3	
17:70159110–70715136	17q24.3	-	P04B_1	P04C_3	
18:77160526–78077248	18q23	-	P04B_1	P04C_1, P04C_3	[21]
20:42723728–47051680	20q13.12, 20q13.13	P04A_9	P04B_3	P04C_1	
22:34700614–35725876	22q12.3	P04A_5, P04A_7, P04A_9	P04B_1	P04C_1	
X:13627396–13995704	Xp22.2	P04A_5	-	P04C_1, P04C_3	
Patient 07					
22:25992468–25992468	22q12.1, 22q12.2, 22q12.3	P07A__1	P07B_3	P07C_2	
Patient 09					
15:60636100–64190630	15q22.2	-	P09B__1	P09C_2	
Patient 10					
16:82480932–85699185	16q23.3, 16q24.1	P10A_1	P10B__1	P10C_1	

Bp: base pair; CTC: circulating tumor cell; ID: identification code; q: long arm; p: short arm.

**Table 3:** List of the enriched terms in CTCs.

Enriched Terms	Term ID	CTCs	Samples	Genes
Natural killer cell activation involved in immune response	GO:0002323	10	P02A__2; P03C_1; P07A__1; P09B__2; P08A__1; P09B__3; P09B__1; P10B_1; P11A_1; P12A_2	*CD244; CORO1A; IFNA1; IFNA10; IFNA14; IFNA16; IFNA17; IFNA2; IFNA21; IFNA4; IFNA5; IFNA6; IFNA7; IFNA8; IFNB1; IFNE; IFNK; IFNW1; KLRF2; VAMP7*
Response to dsRNA	GO:0043331	9	P01A_6; P02A_2; P07A_1; P08A_1; P09B_2; P09B_1; P10B_1; P11A_1; P12A_2	*IFNA1; IFNA10; IFNA14; IFNA16; IFNA17; IFNA2; IFNA21; IFNA4; IFNA5; IFNA6; IFNA7; IFNA8; IFNB1; IFNE; IFNK; IFNW1; IRAK3; PDE12; PMAIP1; RFTN1; RIOK3; SLC3A2; TICAM1; TLR3*
Regulation of peptidyl-serine phosphorylation of STAT protein	GO:0033139	9	P01A_6; P02A_2; P07A_1; P08A_1; P09B_2; P09B_1; P10B_1; P11A_1; P12A_2	*IFNA1; IFNA10; IFNA14; IFNA16; IFNA17; IFNA2; IFNA21; IFNA4; IFNA5; IFNA6; IFNA7; IFNA8; IFNB1; IFNE; IFNG; IFNK; IFNW1; LIF*
Positive regulation of peptidyl-serine phosphorylation of STAT protein	GO:0033141	9	P01A_6; P02A_2; P07A_1; P08A_1; P09B_2; P09B_1; P10B_1; P11A_1; P12A_2	*IFNA1; IFNA10; IFNA14; IFNA16; IFNA17; IFNA2; IFNA21; IFNA4; IFNA5; IFNA6; IFNA7; IFNA8; IFNB1; IFNE; IFNG; IFNK; IFNW1; LIF*
Lymphocyte activation involved in immune response	GO:0002285	9	P01A_6; P02A_2; P07A_1; P08A_1; P09B_2; P09B_1; P10B_1; P11A_1; P12A_2	*CD1C; CD244; EIF2AK4; F2RL1; IFNA1; IFNA10; IFNA14; IFNA16; IFNA17; IFNA2; IFNA21; IFNA4; IFNA5; IFNA6; IFNA7; IFNA8; IFNB1; IFNE; IFNK; IFNW1; LCP1*
Natural killer cell activation	GO:0030101	8	P02A_2; P07A_1; P08A_1; P09B_2; P09B_3; P09B_1; P11A_1; P12A_2	*BAG6; CASP8; CD2; CD244; ELF4; HNF1A; IFNA1; IFNA10; IFNA14; IFNA16; IFNA17; IFNA2; IFNA21; IFNA4; IFNA5; IFNA6; IFNA7; IFNA8; IFNB1; IFNE; IFNK; IFNW1; IL12B; IL2; IL21R; KLRK1; NCR1; NCR3; PRDX1; SLAMF7; SNX27; ULBP1; ULBP2; ULBP3*
B cell proliferation	GO:0042100	8	P01A_6; P02A_2; P07A_1; P08A_1; P09B_2; P09B_1; P11A_1; P12A_2	*BCL2; CD40; CD40LG; CD79A; CR2; CTPS1; GAPT; HSPD1; IFNA1; IFNA10; IFNA14; IFNA16; IFNA17; IFNA2; IFNA21; IFNA4; IFNA5; IFNA6; IFNA7; IFNA8; IFNB1; IFNE; IFNK; IFNW1; IL10; LEF1; MEF2C; MS4A1*
Cytidine to uridine editing	GO:0016554	7	P01A_5; P04A9; P07A_1; P07C2; P08A_1; P09B_3; P11A_1	*A1CF; AICDA; APOBEC1; APOBEC2; APOBEC3A; APOBEC3B; APOBEC3C; APOBEC3D; APOBEC3F; APOBEC3G; APOBEC3H*
T cell activation involved in immune response	GO:0002286	7	P01A_6; P07A_1; P08A_1; P09B_2; P09B_1; P11A_1; P12A_2	*CD1C; EIF2AK4; F2RL1; FCER1G; ICAM1; IFNA1; IFNA10; IFNA14; IFNA16; IFNA17; IFNA2; IFNA21; IFNA4; IFNA5; IFNA6; IFNA7; IFNA8; IFNB1; IFNE; IFNK; IFNW1; ITGAL; LCP1; LILRB1; SLC11A1; TNFSF18*
Lymphocyte proliferation	GO:0046651	7	P02A_2; P07A_1; P08A_1; P09B_2; P09B_1; P11A_1; P12A_2	*BCL2; CD40; CD40LG; CD79A; CR2; CTPS1; ELF4; HELLS; HSPD1; IFNA1; IFNA10; IFNA14; IFNA16; IFNA17; IFNA2; IFNA21; IFNA4; IFNA5; IFNA6; IFNA7; IFNA8; IFNB1; IFNE; IFNK; IFNW1; IL10; MEF2C; MS4A1; MSN; PIK3CG; PPP3CB; TNFRSF4; TNFSF14*
Response to exogenous dsRNA	GO:0043330	7	P01A_6; P07A_1; P08A_1; P09B_2; P09B_1; P11A_1; P12A_2	*CAV1; COLEC12; DDX58; DHX9; FLOT1; IFIH1; IFIT1; IFNA1; IFNA10; IFNA14; IFNA16; IFNA17; IFNA2; IFNA21; IFNA4; IFNA5; IFNA6; IFNA7; IFNA8; IFNB1; IFNE; IFNK; IFNW1; IRAK3; MUL1; PQBP1; RALB; RFTN1; SLC3A2; TICAM1; TLR3; TMEM173*
DNA cytosine deamination	GO:0070383	6	P01A_5; P04A9; P07A_1; P07C2 P08A_1; P11A_1	*APOBEC3A; APOBEC3C; APOBEC3D; APOBEC3F; APOBEC3G; APOBEC3H*
B cell differentiation	GO:0030183	6	P01A_6; P07A_1; P08A_1; P09B_1; P11A_1; P12A_2	*ADAM17; AICDA; BLNK; CD79A; CEBPG; CLCF1; DNAJB9; HDAC5; HDAC9; HHEX; IFNA1; IFNA10; IFNA14; IFNA16; IFNA17; IFNA2; IFNA21; IFNA4; IFNA5; IFNA6; IFNA7; IFNA8; IFNB1; IFNE; IFNK; IFNW1; IL11; ITGA4; JAK3; KIT; KLF6; LYL1; MSH2; NFAM1; NHEJ1; PLCG2; RAG1; RAG2; TCF3; TPD52; VCAM1*
Regulation of type I interferon-mediated signaling pathway	GO:0060338	6	P07A_1; P08A_1; P09B_2; P09B_1; P11A_1; P12A_2	*CACTIN; CDC37; FADD; HSP90AB1; IFNA1; IFNA10; IFNA14; IFNA16; IFNA17; IFNA2; IFNA21; IFNA4; IFNA5; IFNA6; IFNA7; IFNA8; IFNAR1; IFNAR2; IFNB1; JAK1; NLRC5; PTPN1; PTPN11; PTPN6; TYK2; WNT5A; ZBP1*

ID: identification code of the term; CTC: circulating tumor cell; GO: gene onthology; dsRNA: double strand RNA; STAT: Signal Transducer and Activator of Transcription.

## Supplementary Materials

The following are available online at https://www.mdpi.com/2072-6694/12/9/2490/s1, Table S1: CNAs shared between CTCs and matched tumor tissue in the whole case series, Table S2: Enrichment analyses results, Table S3: Clinical-pathological characteristics of patients enrolled in this study.
