# Supplementary material for: Single-Cell NGS-Based Analysis of Copy Number Alterations Reveals New Insights in Circulating Tumor Cells Persistence in Early-Stage Breast Cancer
Source: Cancers (Basel). 2020 Sep 2;12(9):2490. doi: 10.3390/cancers12092490 (PMC7565733; doi:10.3390/cancers12092490)
Supplement: Supplementary file 1 [file cancers-12-02490-s001.zip › cancers-893093 supplementary.docx]

**Supplementary Materials:**

Single-Cell NGS-Based Analysis of Copy Number Alterations Reveals New Insights in Circulating Tumor Cells Persistence in Early-Stage Breast Cancer

**Table S1.** shared between CTCs and matched tumor tissue in the whole case series.

**Table S2:** Enrichment analyses results.

**Table S3.** Clinical-pathological characteristics of patients enrolled in this study. Tumor staging was done based on the tumor (T) and lymph node (N) system.

| **Parameters** | **Patients (*n* = 11)** | | |
| --- | --- | --- | --- |
|  |  | ***n*** | **%** |
| Subtype |  |  |  |
|  | TNBC | 4 | 36.4 |
|  | Luminal-A | 6 | 54.5 |
|  | HER2 enriched | 1 | 9.1 |
| G |  |  |  |
|  | 1 | 1 | 18.2 |
|  | 2 | 4 | 18.2 |
|  | 3 | 6 | 54.5 |
| T |  |  |  |
|  | 1 | 9 | 81.8 |
|  | 2 | 2 | 18.2 |
| N |  |  |  |
|  | 0 | 7 | 63.6 |
|  | 1 | 4 | 36.4 |
| Adjuvant therapy |  |  |  |
|  | C | 3 | 27.3 |
|  | C + Herceptin | 1 | 9.1 |
|  | AI | 2 | 18.2 |
|  | H | 4 | 36.3 |
|  | N/A | 1 | 9.1 |

TNBC: triple negative breast cancer; HER2: Human Epidermal Growth Factor Receptor 2; G: grade; C: chemotherapy; AI: aromatase inhibitor; H: hormone therapy; N/A: not available.
